# Novel Clinical Risk Scoring Model for Predicting Amputation in Patients With Necrotizing Fasciitis: The ANF Risk Scoring System

**DOI:** 10.3389/fmed.2021.719830

**Published:** 2021-11-19

**Authors:** Natthaya Chaomuang, Patcharin Khamnuan, Nipaporn Chuayunan, Acharaporn Duangjai, Surasak Saokaew, Pochamana Phisalprapa

**Affiliations:** ^1^Unit of Excellence on Clinical Outcomes Research and IntegratioN (UNICORN), School of Pharmaceutical Sciences, University of Phayao, Phayao, Thailand; ^2^Division of Pharmacy Practice, Department of Pharmaceutical Care, School of Pharmaceutical Sciences, University of Phayao, Phayao, Thailand; ^3^Center of Health Outcomes Research and Therapeutic Safety (Cohorts), School of Pharmaceutical Sciences, University of Phayao, Phayao, Thailand; ^4^Department of Nursing, Phayao Hospital, Phayao, Thailand; ^5^Unit of Excellence on Herbal Medicine, School of Pharmaceutical Sciences, University of Phayao, Phayao, Thailand; ^6^Biofunctional Molecule Exploratory Research Group, Biomedicine Research Advancement Centre, School of Pharmacy, Monash University Malaysia, Bandar Sunway, Malaysia; ^7^Novel Bacteria and Drug Discovery Research Group, Microbiome and Bioresource Research Strength, Jeffrey Cheah School of Medicine and Health Sciences, Monash University Malaysia, Bandar Sunway, Malaysia; ^8^Department of Physiology, School of Medical Sciences, University of Phayao, Phayao, Thailand; ^9^Division of Ambulatory Medicine, Department of Medicine, Faculty of Medicine Siriraj Hospital, Mahidol University, Bangkok, Thailand

**Keywords:** clinical risk scoring model, amputation, patients, necrotizing fasciitis, ANF risk scoring system

## Abstract

**Background:** Necrotizing fasciitis (NF) is a life-threatening infection of the skin and soft tissue that spreads quickly and requires immediate surgery and medical treatment. Amputation or radical debridement of necrotic tissue is generally always required. The risks and benefits of both the surgical options are weighed before deciding whether to amputate or debride. This study set forth to create an easy-to-use risk scoring system for predicting the risk scoring system for amputation in patients with NF (ANF).

**Methods:** This retrospective study included 1,506 patients diagnosed with surgically confirmed NF at three general hospitals in Thailand from January 2009 to December 2012. All diagnoses were made by surgeons who strictly observed the guidelines for skin and soft tissue infections produced by the Infectious Diseases Society of America. Patients were randomly allocated to either the derivation (*n* = 1,193) or validation (*n* = 313) cohort. Clinical risk factors assessed at the time of recruitment were used to create the risk score, which was then developed using logistic regression. The regression coefficients were converted into item scores, and the total score was calculated.

**Results:** The following four clinical predictors were used to create the model: female gender, diabetes mellitus, wound appearance stage 3 (skin necrosis and gangrene), and creatinine ≥1.6 mg/dL. Using the area under the receiver operating characteristic curve (AuROC), the ANF system showed moderate power (78.68%) to predict amputation in patients with NF with excellent calibration (Hosmer-Lemeshow χ^2^ = 2.59; *p* = 0.8586). The positive likelihood ratio of amputation in low-risk (score ≤ 4) and high-risk (score ≥ 7) patients was 2.17 (95%CI: 1.66–2.82) and 6.18 (95%CI: 4.08–9.36), respectively. The ANF system showed good performance (AuROC 76.82%) when applied in the validation cohort.

**Conclusion:** The developed ANF risk scoring system, which includes four easy to obtain predictors, provides physicians with prediction indices for amputation in patients with NF. This model will assist clinicians with surgical decision-making in this time-sensitive clinical setting.

## Introduction

Necrotizing fasciitis (NF) is a dangerous infection that causes widespread inflammation and necrosis of the soft tissues, and that most often affects the fascia and subcutaneous layers (1). The recommended treatments for patients with NF are emergency surgical debridement and broad-spectrum antibiotic therapy (2). Delayed treatment may lead to significant soft tissue loss and/or limb loss, as well as an increased risk of mortality (3, 4). Patients can experience severe morbidity, such as amputation and organ failure, even after receiving intensive treatment (3, 5, 6). To save the life of a patient, an amputation or radical debridement of necrotic tissue is frequently needed (7). The risks and benefits of each operation are weighed before deciding whether to amputate or dramatically debride (8). NF is rare with a global incidence of 0.3/100,000 per year, and it has been shown that timely diagnosis and surgical management can enhance patient outcomes (9). The fatality and amputation rates associated with NF were estimated to be 15–29% (3, 5) and 20.3–26% (6, 10), respectively. The registered amputation and mortality rates associated with NF in Thailand are 8.7–15.4% and 5.9–22.1%, respectively (11, 12).

The microbes, risk factors, and causes associated with NF have been established, and classification systems have been developed to identify and score patients at high risk for NF (13–17). In 2004, the Laboratory Risk Indicator for NF (LRINEC) score was created for diagnostic NF (18). Among the models that have been created to classify and/or score a patient's risk of NF (14–16), no study has developed a risk-prediction scoring system for amputation in NF.

Accordingly, the aim of this study was to use the epidemiology of NF disease in Thailand to create an easy-to-use risk scoring system for predicting the risk scoring system for amputation in patients with NF (ANF). That score could be used in a routine clinical practice, and it will help physicians and medical team to use it for making decisions on patient care. In addition, our ultimate goal is to help a patient avoid amputation by early prediction and managing the factors related to disease progression.

## Materials and Methods

### Study Design and Study Size

This was a retrospective cohort study. A prognostic prediction analysis and clinical score development study was conducted in accordance with the transparent reporting of a multivariable prediction model for individual prognosis or diagnosis (TRIPOD) statement (19). Ten outcome events per predictor component were used to determine the sample size (the EPV method) (20). Based on other similar scoring systems that collectively draw upon 10 variables, a total of 100 amputations NF were needed.

### Setting and Study Population

From January 2009 to December 2012, the medical records of patients with surgically-confirmed NF were obtained from three general hospitals in Northern Thailand. The hospitals were Chiangrai Prachanukroh Hospital (a 600-bed tertiary-care medical center), Kamphaeng Phet Hospital (a 330-bed public hospital), and Phayao Hospital (a 400-bed secondary-care medical center).

In total, 1,506 patients with NF were enrolled. They were divided into two groups using random sampling by computer generation (4:1), Stata command “*insample*.” The derivation cohort (*n* = 1,193) is a sample to develop ANF scoring system, and a validation cohort (*n* = 313) is to confirm accuracy and appropriateness of this scoring system. In our study, an NF with amputation was found in 127 patients (8.4%). This detail is shown in Table 1.

**Table 1:** Clinical and demographic characteristics of patients with necrotizing fasciitis among all patients, and compared between the derivation cohort and the validation cohort.

**Characteristics**	**All patients (*N* = 1,506)**	**Derivation cohort (*n* = 1,193)**	**Validation cohort (*n* = 313)**
Gender			
Male	848 (56.31%)	686 (57.50%)	162 (51.76%)
Female	658 (43.69%)	507 (42.50%)	151 (48.24%)
Age (years)			
<60	691 (46.25%)	555 (46.91%)	136 (43.73%)
≥60	803 (53.75%)	628 (53.09%)	175 (56.27%)
Body mass index (kg/m^2^)			
≤18.50	197 (14.71%)	156 (14.83%)	41 (14.29%)
18.51–22.99	575 (42.94%)	443 (42.11%)	132 (45.99%)
≥23.00	567 (42.35%)	453 (43.06%)	114 (39.72%)
Education			
No education	652 (43.35%)	524 (43.92%)	128 (41.16%)
Primary education	763 (50.73%)	598 (50.13%)	165 (53.05%)
Secondary education	62 (4.12%)	48 (4.02%)	14 (4.50%)
Bachelor's degree or higher	27 (1.80%)	23 (1.93%)	4 (1.29%)
Occupation			
Elderly living at home	699 (46.41%)	555 (46.52%)	144 (46.01%)
Farmer/laborer	733 (48.67%)	574 (48.11%)	159 (50.80%)
Official	74 (4.91%)	64 (5.36%)	10 (3.19%)
Underlying comorbidities			
Diabetes	387 (25.70%)	305 (25.54%)	82 (26.28%)
Heart disease	96 (6.38%)	74 (6.21%)	22 (7.05%)
Renal disease	45 (2.99%)	39 (3.27 %)	6 (1.92%)
Cirrhosis	61 (4.05%)	55 (4.61%)	6 (1.92%)
Hypertension	538 (35.70%)	434 (36.35%)	104 (33.23%)
Gout	147 (9.75%)	116 (9.72%)	31 (9.90%)
Chronic alcoholism	232 (15.39%)	192 (16.08%)	40 (12.78%)
Wound appearance			
Stage 1 (swelling and erythema)	1,429 (94.82%)	669 (56.08%)	291 (92.97%)
Stage 2 (bleb)	651 (43.20%)	515 (43.17%)	136 (43.45%)
Stage 3 (necrosis and gangrene)	430 (28.53%)	348 (29.17%)	81 (25.88%)
Severe pain	1,316 (87.38%)	1,045 (87.52%)	271 (86.86%)
Sites of wound			
Head and neck	8 (0.53%)	8 (0.67%)	0 (0.0%)
Trunk	28 (1.86%)	26 (2.18%)	2 (0.64%)
Upper limb	276 (18.31%)	219 (18.34%)	57 (18.21%)
Lower limb	1,161 (77.04%)	913 (76.47%)	248 (79.23%)
Fournier's gangrene	29 (1.92%)	23 (1.93%)	6 (1.92%)
Multiple sites	5 (0.33%)	5 (0.42%)	0 (0.0%)
Hospitals			
Chiangrai Prachanukroh	817 (54.21%)	649 (54.36%)	168 (53.67%)
Kamphaeng Phet	557 (36.96%)	429 (35.93%)	128 (40.89%)
Phayao	133 (8.83%)	116 (9.72%)	17 (5.43%)
Laboratory on admission			
WBC (/mm^3^)	16,903.28 ± 236.53	16,783.53 ± 253.48	17,357.72 ± 601.03
PMN (%)	82.05 ± 0.32	81.96 ± 0.37	82.41 ± 0.61
Creatinine (mg/dL)	1.92 ± 0.03	1.95 ± 0.04	1.80 ± 0.08
Bicarbonate (mmol/L)	21.68 ± 0.20	21.51 ± 0.22	22.27 ± 0.41
Total protein (g/dL)	6.33 ± 0.04	6.36 ± 0.05	6.25 ± 0.09
Laboratory 48–72 h			
WBC (/mm3)	14,515.36 ± 356.47	14,132.02 ± 367.71	16,256.68 ± 1,041.54
PMN (%)	78.71 ± 0.64	78.44 ± 0.69	79.95 ± 1.73
Creatinine (mg/dL)	2.25 ± 0.08	2.26 ± 0.09	2.23 ± 0.22
Bicarbonate (mmol/L)	22.51 ± 1.33	23.09 ± 1.67	20.47 ± 1.24
Total protein (g/dL)	5.63 ± 0.11	5.62 ± 0.13	5.67 ± 0.17
Vital signs on admission			
Body temperature (°C)	37.31 ± 0.02	37.30 ± 0.02	37.34 ± 0.04
Pulse rate (/min)	91.40 ± 0.41	91.62 ± 0.46	90.55 ± 0.92
Respiratory rate (/min)	20.15 ± 0.09	20.12 ± 0.10	20.30 ± 0.22
Systolic blood pressure (mmHg)	117.16 ± 0.62	117.36 ± 0.70	116.41 ± 1.38
Diastolic blood pressure (mmHg)	70.21 ± 0.38	70.22 ± 0.42	70.20 ± 0.84
Vital signs 48–72 h			
Body temperature (°C)	37.26 ± 0.02	37.26 ± 0.02	37.28 ± 0.04
Pulse rate (/min)	87.74 ± 0.38	87.42 ± 0.42	88.94 ± 0.87
Respiratory rate (/min)	19.46 ± 0.11	19.47 ± 0.12	19.43 ± 0.26
Systolic blood pressure (mmHg)	120.69 ± 0.47	120.81 ± 0.55	120.26 ± 0.95
Diastolic blood pressure (mmHg)	73.10 ± 0.30	72.95 ± 0.34	73.64 ± 0.65
Amputation	127 (8.4%)	99 (8.3%)	28 (8.95 %)
Site of amputation			
Finger/toes	58 (45.67%)	47 (47.47%)	11 (39.28%)
Above knee	29 (22.83%)	24 (24.24%)	5 (17.85%)
Below knee	26 (20.47%)	19 (19.19%)	7 (25.00%)
Forefoot	11 (8.66%)	7 (7.07 %)	4 (14.28 %)
Hand and forearm	3 (2.36%)	2 (2.02%)	1 (3.57%)

*Data presented as mean ± standard deviation or number and percentage*.

*WBC, white blood cell count; PMN, polymorphonuclear cell or neutrophil*.

Necrotizing fasciitis was defined by the presence of extensive necrosis affecting at least the fascia and subcutaneous tissue. Patients diagnosed with NF according to the standard definition were eligible for inclusion. Diagnoses were made by surgeons who strictly observed the guidelines for skin and soft tissue infections produced by the Infectious Diseases Society of America (21).

The stages of wound appearance were defined as a clinical stage of NF. Cutaneous manifestations of NF present when the disease progresses through stages 1, 2, and 3 (early, intermediate, and late stages). Wound appearance stage 1 (early): the disease was clinically presenting with swelling and erythema; stage 2 (intermediate): belb formation is an important diagnostic clue of NF. When present, it signals the onset of critical skin ischemia; stage 3 (late): indicates the development of tissue necrosis and skin gangrene, which are severe signs of NF infection (21, 22).

Severe pain was defined based on the patient complaint by use of a pain-assessment tool (numerical rating scale 7–10) according to the pain management guideline 2017 (23, 24).

### Ethics Approval

The protocol for this study was approved by the Ethics Committee of Chiangrai Prachanukroh Hospital and the Faculty of Medicine, Chiang Mai University, Chiang Mai, Thailand (032/2013; research ID: 1461; study code: COM-13-1461-EX).

### Statistical Analyses

Descriptive statistics were used to summarize patient demographic and clinical characteristics. Continuous data were compared using Student's *t*-test, and those results are shown as mean ± standard deviation (SD). Categorical data were compared using chi-square test or Fisher's exact test, and those results are given as number and percentage. All *p*-values are two-tailed and were considered statistically significant at a *p*-value < 0.05.

### Model Development

Variables that could affect the outcomes were established and incorporated into the development model. The predictive variables were selected by factors reported from previous studies to ensure a clinical significance (25–27). Clinically meaningful and interpretable cut-off points were used to transform continuous variables into categorical variables. In addition, we also selected the parameters from statistical analysis that *P*-values < 0.05 were considered statistically significant to include in running the model. Amputation and no amputation variables were evaluated using univariable logistic regression, and those results are summarized as odds ratio (OR) and 95% confidence interval (CI). Variables found to be statistically significant and other variables of interest were then entered into a multivariable logistic regression model to identify factors independently associated with amputation in NF. Item scores were calculated using the weighted coefficients of the significant variables from multivariable analysis. By dividing each regression coefficient by the smallest coefficient of the model and rounding to the nearest integer, the score was determined (28–30). To determine the power of the model to discriminate between amputation and no amputation, a receiver operating characteristic (ROC) curve was plotted, and the area under the ROC curve (AuROC) was also calculated. A Hosmer–Lemeshow goodness of fit test was also performed (31). Cut-off scores were determined to classify the patients with NF as low-risk, moderate-risk, or high risk for death. Sensitivity, specificity, positive predictive value (PPV), negative predictive value (NVP), positive likelihood ratio (LR+), and negative likelihood ratio (LR–) were calculated to evaluate the diagnostic performance of the model (32).

### Model Validation

The validation cohort (*n* = 313) was used to verify the scoring system. The efficiency and accuracy of the model were assessed using a ROC curve. The same diagnostic performance parameters that were calculated and assessed in the derivation cohort were calculated and assessed in the validation cohort.

## Results

### Patient Characteristics

A total of 1,506 patients with NF were enrolled. Their ages ranged from 2 to 95 years, 43.69% were female, 85.29% had a body mass index (BMI) ≥18.50, and 25.70% had diabetes mellitus. A swelling wound (wound appearance stage 1) was presented in 94.82% of patients, and the most common wound site was the lower limbs (77.04%). An amputation with NF found 127 patients (8.4%) and non-amputation with NF found 1,379 patients (91.6%). Sites of amputation were finger/toes (44.1%), above knee (22.8%), below knee (20.5%), forefoot (8.7%), hand and forearm (3.9%). Clinical and demographic characteristics of patients with NF among all patients and comparison between the derivation cohort and the validation cohort are shown in Table 1.

### Predictors of Amputation Patients With NF

The overall amputation rate in this study was 8.43% (127 of 1,506 patients). Univariable analysis for risk factors significantly associated with amputation in patients with NF in the derivation cohort is shown in Table 2. Factors found to be significantly associated with amputation were female gender, age, education, heart disease, hypertension, erythematous wound, bleb wound, white blood cell count (WBC), percentage of polymorphonuclear cell or neutrophil, serum creatinine, serum bicarbonate, pulse rate, systolic blood pressure, diastolic blood pressure, severe sepsis, and length of hospital stay.

**Table 2:** Univariable analysis for risk factors significantly associated with amputation in patients with necrotizing fasciitis in the derivation cohort.

**Variables**	**Derivation cohort (*****n*** **=** **1,193)**		
	**Odds ratio**	**95% CI of odds ratio**	***p*-value**
Female gender	1.62	1.05–2.51	***0.0203***
Age (per year) group	1.37	0.88–2.15	0.1381
Body mass index	1.00	0.63–1.60	0.9897
Education	1.33	0.86–2.07	0.1670
Occupation	1.34	0.87–2.09	0.1610
Underlying comorbidities			
Diabetes	4.24	2.72–6.61	***<0.0001***
Heart disease	1.80	0.79–3.69	0.0942
Renal disease	1.65	0.49–4.39	0.2988
Cirrhosis	0.19	0.01–1.17	0.0745
Hypertension	1.32	0.85–2.05	0.1854
Gout	0.80	0.32–1.71	0.5646
Chronic alcoholism	1.77	0.89–3.90	0.0902
Wound appearance			
Stage 1 (swelling and erythema)	1.41	0.90–2.22	0.1135
Stage 2 (bleb)	0.80	0.51–1.25	0.3155
Stage 3 (necrosis and gangrene)	4.31	2.75–6.76	***<0.0001***
Severe pain	0.78	0.43–1.50	0.4028
Sites of wound			
Upper limb	0.71	0.36–1.29	0.2579
Lower limb	1.41	0.82–2.55	0.1948
Multiple sites	2.38	0.57–7.36	0.1104
Laboratory on admission			
WBC (/mm^3^)	1.63	0.75–3.23	0.1457
PMN (%)	0.93	0.60–1.45	0.7665
Creatinine (mg/dL)	2.34	1.49–3.73	***0.0001***
Bicarbonate (mmol/L)	1.47	0.35–4.49	0.4820
Total protein (g/dL)	1.08	0.02–8.01	0.9375
Laboratory 48–72 h			
WBC (/mm^3^)	0.79	0.15– 2.73	0.7148
PMN (%)	1.21	0.60– 2.36	0.5529
Creatinine (mg/dL)	1.35	0.74– 2.49	0.2826
Bicarbonate (mmol/L)	0.00	0.00–3.03	0.2453
Total protein (g/dL)	0.00	0.00–4.27	0.3240
Vital signs on admission			
Body temperature (°C)	1.34	0.84–2.10	0.1769
Pulse rate (/min)	1.10	0.12–4.64	0.8977
Respiratory rate (/min)	2.30	0.36–95.4	0.4026
Systolic blood pressure (mmHg)	0.92	0.39–1.90	0.8294
Diastolic blood pressure (mmHg)	1.08	0.41–2.45	0.8378
Vital signs 48–72 h			
Body temperature (°C)	1.62	0.95– 2.67	0.0488
Pulse rate (/min)	0.00	0.00–3.61	0.3007
Respiratory rate (/min)	0.91	1.42– 1.77	0.7807
Systolic blood pressure (mmHg)	1.28	0.14–5.61	0.7370
Diastolic blood pressure (mmHg)	5.42	1.41–17.79	0.0007

*A p-value < 0.05 indicates statistical significance*.

*WBC, white blood cell count; PMN, polymorphonuclear cell or neutrophil*.

*The bold and italic values mean p value < 0.05 indicates statistic significant*.

### Model Development

The variables identified in univariable analysis were entered into subsequent multivariable analysis to develop the scoring system for amputation in patients with NF. Using backward stepwise logistic regression, the multivariable model included four variables. The weighted coefficient was used to calculate the point value for each factor, which was then rounded to the nearest whole number (Table 3). The total prediction score comprises the summation of the following four scores: female gender (yes = 1, no = 0); diabetes mellitus (yes = 3, no = 0); wound appearance stage 3 (yes = 3, no = 0); and creatinine ≥1.6 mg/dL (yes = 2, no = 0). The highest possible score is 9, and a higher score indicates a higher risk for amputation.

**Table 3:** Multivariable analysis and risk score for amputation in patients with necrotizing fasciitis.

**Predictors**	**Coefficient**	**aOR**	**95% CI of aOR**	***p***	**Assigned score**
Female gender	0.4593086	1.58	1.01–2.47	***0.043***	1
Diabetes mellitus	1.2440160	3.47	2.23–5.41	***<0.001***	3
Wound appearance: stage 3	1.4157790	4.11	2.71–6.25	***<0.001***	3
Creatinine ≥1.6 mg/dL	0.9409972	2.56	1.62–4.05	***<0.001***	2

*p-value < 0.05 indicates statistical significance*.

*aOR, adjusted odds ratio; CI, confidence interval*.

*The bold and italic values mean p value < 0.05 indicates statistic significant*.

**Figure 1:**
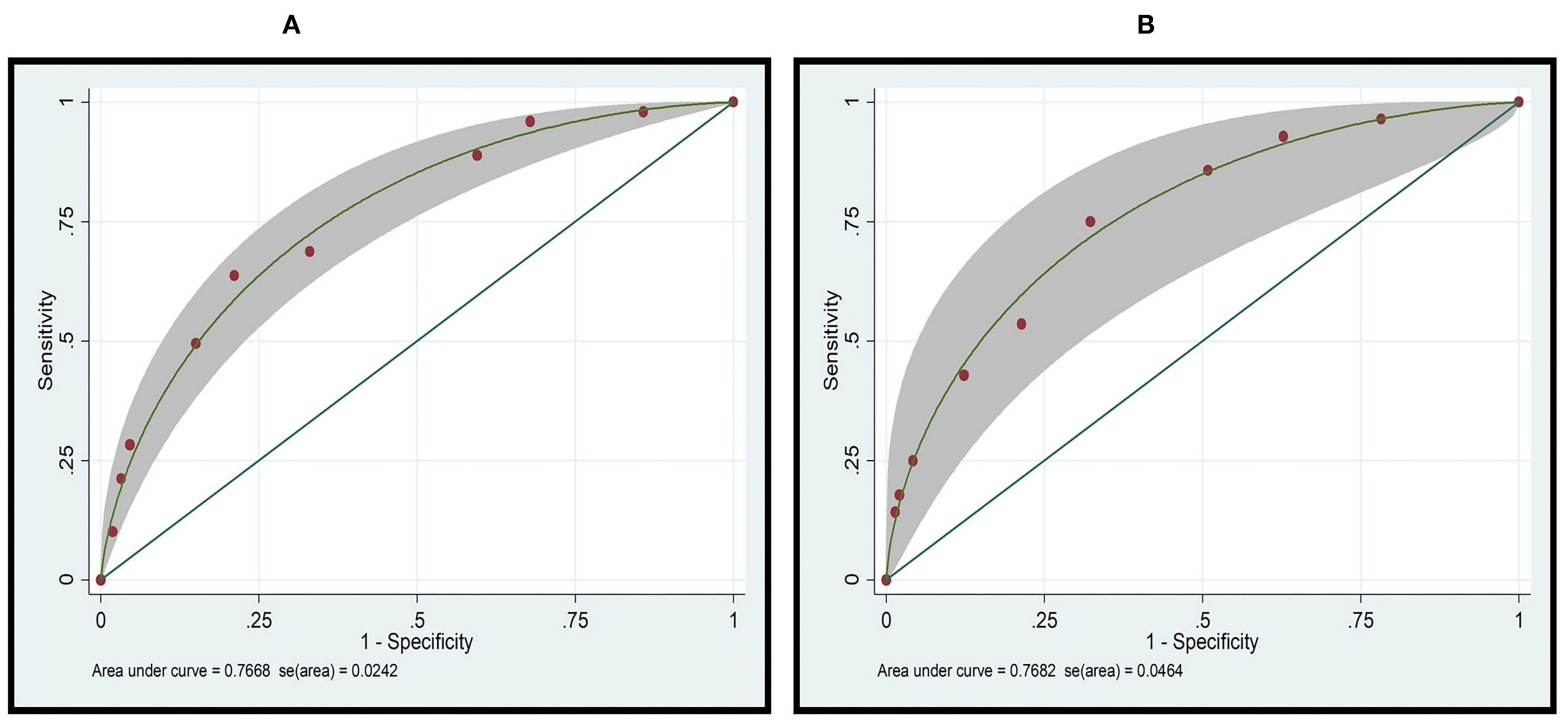
Receiver operator characteristic (ROC) curve analysis of the ANF risk scoring system for predicting amputation in patients with necrotizing fasciitis in the **(A)** derivation cohort (*n* = 1,193), and the **(B)** validation cohort (*n* = 313).

To categorize patients into three risk categories, cut-off scores of 4 and 7 were selected. Patients with a total score of ≥7 were considered to be at high risk. Amputation among those patients was found to be predicted with moderate accuracy (28/78 cases; PPV 35.89%). Patients with a total score of ≤4 were considered to be at low risk. No amputation was correctly predicted in 95.99% of cases (863/899) in this group. No amputation group could be excluded with moderate accuracy (NPV 21.43%). The corrected prediction of absence or presence of amputation was (863 + 28)/(899 + 78) = 91.19%, whereas the incorrect prediction rate was (36 + 50)/(899 + 78) = 8.80% (Table 4). With this scoring system and using two cut-off points, the risk score discriminated between patients with NF with a risk for amputation and those without a risk for amputation with high validity (AuROC 76.68%) (Figure 1). The predictive model was also shown to be well-calibrated (Hosmer-Lemeshow χ^2^ = 2.59; *p* = 0.8586).

**Table 4:** Distribution of risk of amputation in patients with necrotizing fasciitis, diagnostic performance, and interpretation in the derivation cohort (*n* = 1,193).

**Derivation cohort**	**Low risk (score ≤ 4)**	**Moderate risk (score 5–6)**	**High risk (score ≥ 7)**	**Total**
Total	899	216	78	1,193
No amputation	863	181	50	1,094
Amputation	36	35	28	99
Diagnostic performance				
Sensitivity	78.88%		28.28%	
Specificity	63.63%		95.43%	
Positive predictive value	95.99%		35.89%	
Negative predictive value	21.43%		93.63%	
Likelihood ratio (+)	2.17 (95%CI: 1.66**–**2.82)		6.18 (95%CI: 4.08**–**9.36)	
Likelihood ratio (–)	0.33 (95%CI: 0.27**–**0.40)		0.75 (95%CI: 0.66**–**0.85)	

*CI, confidence interval*.

### Model Validation

The ROC curves for the derivation and validation cohorts showed similar results (AuROC 76.68 and 76.82%, respectively; Figure 1). Amputation in the high-risk group was accurately predicted in 36.84% of cases, and the presence of amputation was predicted with high moderate accuracy (PPV 36.84%). In the low-risk group, no amputation was predicted in 94.51% (224/237) of the cases, with the absence of amputation being predicted with moderate accuracy (NPV 34.75%). The accurate prediction of amputation or no amputation was (224 + 7)/(237 + 19) = 90.23%, whereas the incorrect prediction rate was (13 + 12)/(237 + 19) = 9.76% (Table 5).

**Table 5:** Distribution of risk of amputation in patients with necrotizing fasciitis, diagnostic performance, and interpretation in the validation cohort (*n* = 313).

**Validation cohort**	**Low risk (score ≤ 4)**	**Moderate risk (score 5–6)**	**High risk (score ≥ 7)**	**Total**
Total	237	57	19	313
No amputation	224	49	12	285
Amputation	13	8	7	28
Diagnostic performance			
Sensitivity	78.59%		25.00%	
Specificity	53.57%		95.78%	
Positive predictive value	94.51%		36.84%	
Negative predictive value	19.74%		92.86%	
Likelihood ratio (+)	1.69 (95%CI: 1.13**–**2.53)		5.94 (95%CI: 2.54**–**13.85)	
Likelihood ratio (–)	0.39 (95%CI: 0.27**–**0.00)		0.78 (95%CI: 0.63**–**0.97)	

*CI, confidence interval*.

**Figure 2:**
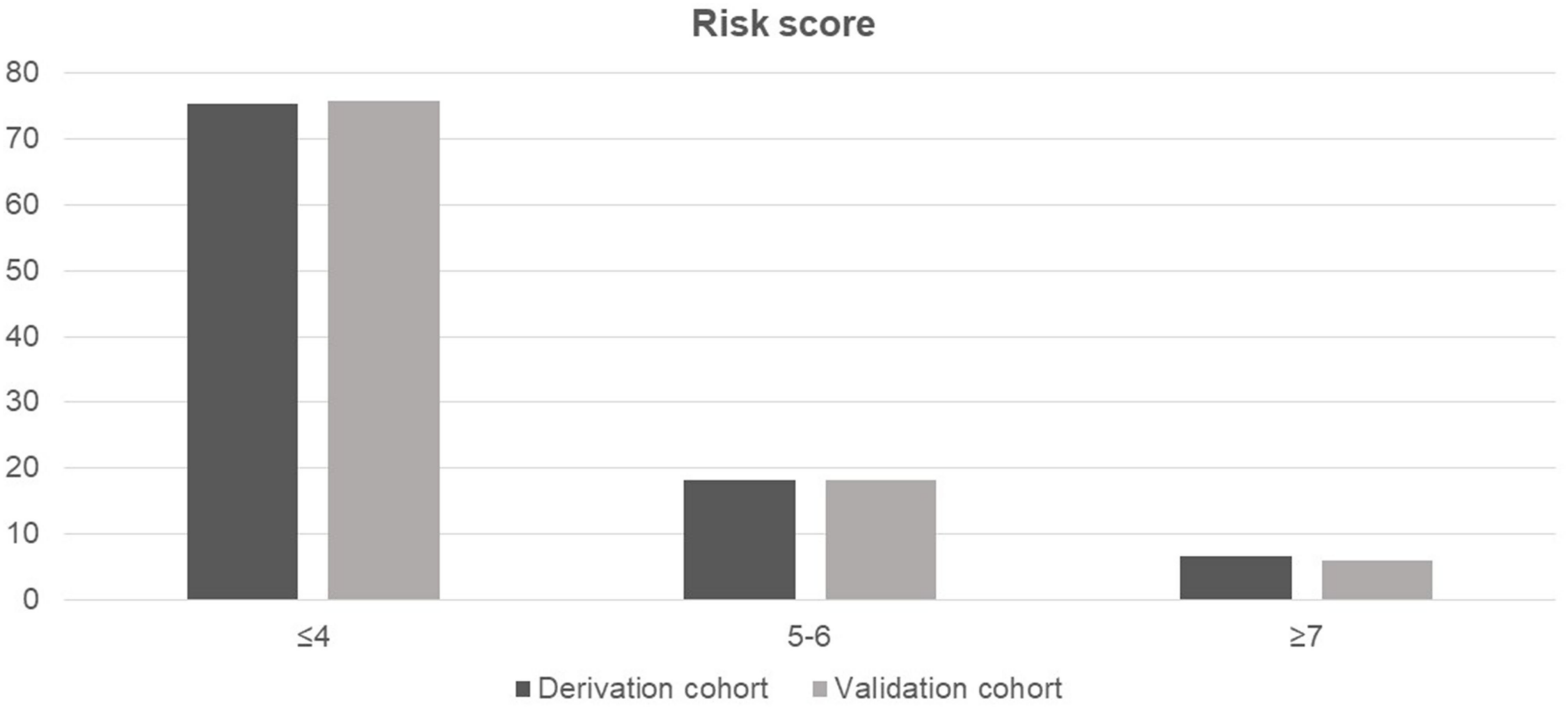
Probability of amputation in patients with necrotizing fasciitis stratified by risk score and compared between the derivation and validation cohorts.

The probability of amputation in patients with NF stratified by risk score and compared between the derivation and validation cohorts is shown in Figure 2. Patients were classified into three groups based on the calculated cut-off values, as follows: low-risk (score ≤4), moderate-risk (score of 5–6), or high-risk (score ≥7). All comparisons among the three risk groups were statistically significant with *p*-values <0.001 for all comparisons in both the cohorts.

## Discussion

In this study, we developed a clinical risk scoring system to predict amputation in patients with NF (the ANF risk scoring system) that can be used as a screening tool to identify NF patients at risk for amputation. The ANF risk scoring system grades NF patients as being at low risk, moderate risk, or high risk for amputation, and this information will help clinicians with treatment decision-making. Amputation is an intensive treatment that can cause significant morbidity and organ failure; however, patients with high mortality risk are often indicated for primary amputation to prevent death. The ANF tool may be useful in decision-making for the clinical management of patients with NF who have been admitted to the hospital.

We divided the patients in the NF patients in the derivation group into the amputation and no amputation groups. We then analyzed for factors that were significantly different between those two groups. Significant factors from univariable analysis were then entered into a multivariable logistic regression model to identify predictors independently associated with amputation. The ANF risk scoring system includes predictors related to a patient demography (female gender), patient comorbidity (diabetes mellitus), clinical sign (wound appearance stage 3, presenting with gangrene and necrosis of the skin), and laboratory result (creatinine ≥1.6 mg/dL). These predictors are similar to the important risk factors for amputation identified by many other studies (27).

The patients with NF in this study were classified into three groups according to their likelihood of amputation (low risk, moderate risk, or high risk). The ANF risk scoring system was internally validated, and it showed high discriminative power when evaluated in this study's validation cohort. The cut-off points used in this study were determined from evaluations of sensitivity, specificity, and positive and negative predictive values. The cut-off point for high risk for amputation was determined to be an ANF score of ≥7. Using this system, patients scoring ≤4 are classified as low-risk, and patients scoring 5–6 are classified as being at moderate risk for amputation. The ANF score of a patient can then be combined with other factors and the judgment of the physician. Patients with an ANF score ≥7 should be closely monitored and managed to avoid limb loss or death, if possible.

The ANF score developed in this study differs from the LRINEC score (14, 15, 18). The tool is based on six laboratory variables at the time of presentation, including C-reactive protein (CRP), total white cell count, hemoglobin, serum sodium, creatinine, and glucose. The LRINEC score is a reliable tool that can aid in the clinical diagnosis of NF (18). It can also identify high-risk patients and predict NF outcome (33). In 2008, Su et al. reported that patients with a LRINEC score of ≥6 had a significantly higher rate of amputation (*P* = 0.002) (16). In contrast, Leiblien et al. reported that a high LRINEC score (≥8; *p* = 0.19) had no significant association with the amputation rate (34). The LRINEC score examines six laboratory-based parameters, but no clinical parameters, such as comorbidities and clinical observations, are taken into consideration (14). In contrast to the LRINEC score, the ANF risk scoring system was developed specifically to predict amputation in patients with NF. We utilize the LRINEC score to predict amputation prognosis in our data. We found the LRINEC score had low sensitivity (51.94 vs. 78.88%) and specificity (92.33 vs. 95.43%) compared with our score (the ANF risk scoring system) in our study.

Women have more subcutaneous fat than men, which makes them more vulnerable to infection. Previous studies have shown that female gender, diabetes, cutaneous gangrene on admission, symptoms of an underlying condition, clostridial infection, heart disease, and shock (systolic blood pressure <90 mmHg) at hospital admission are all independent predictors of limb loss in patients with NF (5, 35).

Diabetes was previously reported to be a clinical predictor of limb amputation in patients with NF. In our study, the prevalence of diabetes was 25.70%. Our finding that diabetes is independently associated with limb loss is similar to the reported findings of several previous studies (5, 34, 36, 37). Hyperglycemia causes bacterial growth and tissue ischemia as a consequence of peripheral vascular disease. Diabetes is, therefore, associated with worse outcomes in patients with NF, including amputation. Atherosclerosis is also common in diabetic patients. Small-caliber arteries were found to have more severe atherosclerotic changes in limb vessels, resulting in ischemia and gangrene, and eventually amputation (36, 37).

Wang et al. developed a classification of wound appearance stages based on clinical symptoms that facilitates rapid diagnosis and treatment (22). In the early stages of infection, this may include tenderness to palpation that extends beyond the visible region of skin involvement. Capillary leakage and elevated temperature cause erythema and swelling/edema. Blister and bullae formation are essential symptoms of skin ischemia in intermediate stage 2. Crepitus, skin anesthesia, and necrosis with a dusky discoloration occur in stage 3 (22). Wound appearance stage 3 with skin necrosis and gangrene was found to be a predictor of amputation in our research. Khamnuan et al. reported NF presenting with gangrene and skin necrosis at the time of diagnosis to be a strong predictor of amputation (*p* < 0.001) (27). Similarly, previous studies reported cutaneous gangrene presented on admission to be significantly associated with a higher risk of amputation (*p* = 0.005) (4, 6, 7, 38). Skin necrosis and gangrene were the most common causes of poor prognosis in late-stage NF cases (22). In most cases with NF, soft tissue swelling in the affected area was a common clinical symptom. Tissue edema and muscle necrosis are caused by infection that has spread across the lymphatic and vascular systems. Skin necrosis is caused by thrombosis of microvascular vessels and nerve supply dysfunction. A loss of blood flow causes gangrene, which is necrosis of the tissue. Depending on the extent of the necrotic areas, debridement or amputation was required whenever gangrene was present (26, 39).

A higher risk of limb failure was found in our sample when the serum creatinine level was >1.6 mg/dL. Renal dysfunction, which is thought to be linked with septic shock, is reflected by a higher serum creatinine level. Several previous studies reported shock (systolic blood pressure <90 mmHg) at admission to be an independent predictor of limb loss, elevated creatinine level, and higher mortality (40).

### Strengths

The strengths of this study should be emphasized. First, this study included a large sample size of patients with NF to evaluate amputation outcome. We also included patients from three large hospitals in Thailand, which suggests that our results can be generalized to other regions of Thailand, and to other low to middle income Asian countries. Second, the ANF risk scoring system was developed in accordance with the stringent criteria set forth in the TRIPOD statement (19). Third, the developed scoring system comprises only four variables, and all of them are easy to obtain and quantify. Interestingly, the predictors that were identified are different types of parameters. ANF scoring includes patient demographic data (being female), patient comorbidity data (diabetes mellitus), patient clinical characteristic data (wound appearance stage 3, presenting with skin necrosis and gangrene), and patient routine laboratory data (serum creatinine). Fourth, the ANF risk scoring system was validated using different patient data sets. In both the derivation and validation cohorts, the ANF score demonstrated good prediction ability and satisfactory diagnostic results. Fifth and last, since serum creatinine is the only laboratory test that is needed, this model is very affordable. The cost of this investigation is only 60 Thai baht (THB) (2 USD), and serum creatinine is a routine laboratory test that is routinely used to track patient health status while they are in the hospital.

### Limitations

The most notable limitation of this study is it retrospective design, which rendered it vulnerable to missing or incomplete data. For example, the LRINEC scoring system could not be used in this study because some important laboratory investigations, such as CRP level, were not performed in all patients.

The data on the amputations index-date, time, and duration of index-operations were not collected because of the retrospective analysis from January 2009 to December 2012. However, since the reason of performing an amputation is to control the infection indicated by several outcomes, e.g., reducing length-of-stay (LOS) or mortality rate, we found that LOS in a hospital of non-amputated is significantly lower (11 days, SD = 11.59) than those in amputated groups (16 days, SD = 10.79), *p* < 0.001. However, there was no difference in mortality between amputations and no amputations (20.20 vs. 19.12 %, Odds ratio 1.07: 95% Cl 0.61–1.81). The data on the amputations index date, time, and duration of index operations were not collected. However, we have the data of length of stay in a hospital of non-amputated, which was 11 days (SD = 11.59) and in amputated it was 16 days (SD = 10.79), with a *P*-value < 0.001. Amputation was performed to control the infection. However, there was no difference in mortality between amputations and no amputations. Our data showed that the mortality of amputated was 20.20% and non-amputated was 19.12% (Odds ratio 1.07; 95% Cl 0.61–1.81). This was similar to previous studies (1, 26) which reported that amputation did not show the reduction of mortality, but patients who underwent amputation had to undergo fewer operations to control the infection and to achieve wound coverage. Kaplan–Meier survivorship analysis revealed that the survival rate decreased with delayed surgery and prolonged symptoms. Survival sharply declined with a delay in surgery of more than 24 h.

Despite this limitation, the ANF risk scoring system can be used in all clinical settings because of its low-cost and because it is simple to implement and use. The advantage of the ANF score is that it can help to detect early the amputation risk in patients with NF. Using this system, disease progression can be stopped or reduced, potential complications of NF can be monitored, and the risk of amputation can be greatly reduced or eliminated.

### Conclusion

The developed ANF risk scoring system, which includes four easy to obtain predictors, provides physicians with prediction indices for amputation in patients with NF. This model will assist clinicians with surgical decision-making in this time-sensitive clinical setting.

## Data Availability Statement

The original contributions presented in the study are included in the article/Supplementary Materials, further inquiries can be directed to the corresponding authors at: coco_a105@hotmail.com.

## Ethics Statement

The studies involving human participants were reviewed and approved by the Ethics Committee of Chiangrai Prachanukroh Hospital and the Faculty of Medicine, Chiang Mai University, Chiang Mai, Thailand (032/2013; research ID: 1461; study code: COM-13-1461-EX). Written informed consent for participation was not required for this study in accordance with the national legislation and the institutional requirements.

## Author Contributions

NCha, PK, and SS: study concept and design, statistical analysis, and interpretation of data. PK, NChu, and SS: acquisition of data. NCha, SS, and PP: drafting of the manuscript. NCha, PK, NChu, AD, SS, and PP: critical revision of the manuscript. All authors contributed to the article and approved the submitted version.

## Funding

This study was supported by a grant from the University of Phayao *via* the Unit of Excellence on Clinical Outcomes Research and IntegratioN (UNICORN).

## Conflict of Interest

The authors declare that the research was conducted in the absence of any commercial or financial relationships that could be construed as a potential conflict of interest.

## Publisher's Note

All claims expressed in this article are solely those of the authors and do not necessarily represent those of their affiliated organizations, or those of the publisher, the editors and the reviewers. Any product that may be evaluated in this article, or claim that may be made by its manufacturer, is not guaranteed or endorsed by the publisher.
